# Updates on HIPK2: a resourceful oncosuppressor for clearing cancer

**DOI:** 10.1186/1756-9966-31-63

**Published:** 2012-08-13

**Authors:** Gabriella D’Orazi, Cinzia Rinaldo, Silvia Soddu

**Affiliations:** 1 Department of Medical, Oral, and Biotechnological Sciences, University “G. d’Annunzio”, Chieti 66013, Italy; 2 Department of Experimental Oncology, Regina Elena National Cancer Institute, Rome 00158, Italy; 3 Institute of Molecular Biology and Pathology, National Research Council (CNR), c/o Sapienza University of Rome, Rome 00185, Italy

**Keywords:** HIPK2, Oncosuppressor p53, p53-family members, Apoptosis, Genotoxic damage, Hypoxia, Tumorigenesis, siRNA interference, Gene knockout, Cytokinesis

## Abstract

Homeodomain-interacting protein kinase 2 (HIPK2) is a multitalented protein that exploits its kinase activity to modulate key molecular pathways in cancer to restrain tumor growth and induce response to therapies. HIPK2 phosphorylates oncosuppressor p53 for apoptotic activation. In addition, also p53-independent apoptotic pathways are regulated by HIPK2 and can be exploited for anticancer purpose too. Therefore, HIPK2 activity is considered a central switch in targeting tumor cells toward apoptosis upon genotoxic damage and the preservation and/or restoration of HIPK2 function is crucial for an efficient tumor response to therapies. As a proof of principle, HIPK2 knockdown impairs p53 function, induces chemoresistance, angiogenesis, and tumor growth *in vivo*, on the contrary, HIPK2 overexpression activates apoptotic pathways, counteracts hypoxia, inhibits angiogenesis, and induces chemosensitivity both in p53-dependent and -independent ways. The role of HIPK2 in restraining tumor development was also confirmed by studies with HIPK2 knockout mice. Recent findings demonstrated that HIPK2 inhibitions do exist in tumors and depend by several mechanisms including HIPK2 cytoplasmic localization, protein degradation, and loss of heterozygosity (LOH), recapitulating the biological outcome obtained by RNA interference studies in tumor cells, such as p53 inactivation, resistance to therapies, apoptosis inhibition, and tumor progression. These findings may lead to new diagnostic and therapeutic approaches for treating cancer patients. This review will focus on the last updates about HIPK2 contribution in tumorigenesis and cancer treatment.

## Introduction

An outstanding problem in cancer therapy is the battle against treatment-resistant disease. Several genetic and epigenetic conditions as well as microenvironment modifications, contribute to tumor resistance to therapies, including p53 inactivation, induction of hypoxia, immunosuppression, and DNA repair [1]. One of the most promising molecules that might be exploited in anticancer therapy is homeodomain-interacting protein kinase 2 (HIPK2). HIPK2 has been discovered more than 10 years ago as a nuclear serine/threonine kinase that acts as corepressor for transcription factors [2]. Since then, HIPK2 has been shown to regulate gene expression, during DNA damage response and development, by phosphorylating a ever growing number of transcription factors and by recruiting corepressor components involved in gene transcription, as summarized in several reviewss [3,4]. HIPK2 function is important in anticancer therapy because it induces tumor cell apoptosis, an outcome obtained by activating various downstream signaling pathways [5], most prominently oncosuppressor p53 [6]. HIPK2 may induce apoptosis also by modulating molecules independently by p53, such as through phosphorylation-dependent degradation of anti-apoptotic transcriptional corepressor CtBP [7], underlying its role as regulator of several different molecules.

The p53 tumor suppressor is a zinc-protein that is activated in response to DNA damage [8]. The function of p53 as a tumor suppressor is linked to its activity as transcription factor through posttranslational modifications that allow the protein to bind DNA and induce target genes (encoding both proteins and microRNA) involved in cell-cycle arrest, senescence, and apoptosis [9]. Given its crucial role as “guardian of the genome”, tumors press to inactivate p53 at different tumor stages through several mechanisms including gene mutations, protein inactivation, or inactivation of p53 regulatory proteins [10]. Impairment of p53 function has a crucial role in tumor evolution by allowing evasion from p53-dependent responses. Therefore, restoration of p53 activity in tumor cells is a valuable intervention for tumor regression [11]. Recent studies from our groups and others’ have shed new lights on various aspects of p53 regulation by HIPK2 and have served to both increase the complexity of the p53 regulatory pathways, including p53 inhibitors (*i.e.*, MDM2) and p53-family members (*i.e.*, ΔNp63α) but also to underline a role for HIPK2 as tumor suppressor itself for anticancer therapy, that we will discuss here. Thus, HIPK2 inactivation unlashes signaling pathways that lead to p53 dysfunction, chemoresistance, angiogenesis and tumor growth [12,13]. For these reasons, HIPK2 is a promising biomarker and a target for tumor therapy. Understanding the molecular mechanisms underlying HIPK2 activation and inactivation will therefore give more insight into its role in tumor development and regression.

### HIPK2 activates p53 apoptotic function in response to genotoxic stress

HIPK2 can be activated by several types of genotoxic damage, including ultraviolet radiation (UV), ionizing radiation (IR), and antitumor drugs such as cisplatin (CDDP), adriamycin (ADR) and roscovitin [6,14-16]. One of the main molecules activated by HIPK2 is the p53 oncosuppressor. HIPK2 phosphorylates p53 at serine 46 (Ser46) [6] and allows recruitment of histone acetylase (HAT) p300 for efficient p53 acetylation at lysine 382 (Lys382) [17]. These p53 posttranslational modifications specifically induce p53-dependent pro-apoptotic gene transcription (*i.e.*, p53AIP1, Noxa, Puma, Bax, Killer DR5) while p53 regulatory genes such as MDM2 or cell-cycle arrest related p21 are not induced [12,18]. Interestingly, p53 activation induces caspase-6 which is responsible for caspase-mediated HIPK2 cleavage at positions 916 and 977 [19]. This C-terminus truncated HIPK2 results in a hyperactive kinase which potentiates p53Ser46 phosphorylation and activation of apoptosis and eventually is degraded. Thus, caspase-resistant HIPK2 mutants induce apoptosis less efficiently than wild-type [19]. These findings suggest a tight regulation of HIPK2 in a p53-dependent manner, a regulatory loop similar to the elimination of ERK2 kinase by a p53-induced apoptotic program, in order to prevent ERK-mediated cell proliferation in the presence of activated p53 [20].

HIPK2 is a critical activator of p53 function in response to drugs as substantiate by experiments of HIPK2 gene silencing by small interference RNA (siRNA). HIPK2 knockdown impairs p53 pro-apoptotic gene transcription in response to drugs and predisposes to chemoresistance [14] and increased tumor growth *in vivo*[21]. HIPK2 knockdown contributes to p53 inactivation by different means other than by direct impairment of p53Ser46 phosphorylation. cDNA microarray of colon cancer cells with chronic depletion of HIPK2 function by siRNA [22], showed upregulation of two novel targets of HIPK2 corepressor function that are involved in p53 deregulation, that is, Nox1 and MT2A. Thus, HIPK2 has been shown to repress Nox1 promoter activity [23]. Nox1 is a homolog of the catalytic subunit of the superoxide-generating NADPH-oxidase that is often overexpressed in tumors and is involved in tumor progression and angiogenesis [24]. HIPK2 knockdown induces Nox1 upregulation and Nox1 overexpression impairs p53 apoptotic transcriptional activity by inducing p53Lys382 deacetylation [23].

Interestingly, chronic HIPK2 depletion leads to p53 protein misfolding, as assessed by immunoprecipitation studies with conformation-specific p53 antibodies, that impairs p53/DNA binding and p53 transcriptional activity [22]. This p53 misfolding, in colon and breast cancer cells, could be, at least in part, ascribed to metallothionein 2A (MT2A) upregulation upon HIPK2 depletion [25]. Thus, MT2A depletion by siRNA, restores wtp53 native conformation and p53 function in response to drugs, in HIPK2 knockdown cells [25]. Metallothionein is a family of at least 10 conserved isoforms of metal-binding cysteine-rich proteins with a potential role in homeostasis of essential metals [26]. MTs upregulation has been found in several human tumors including breast, colon, liver, and lung, and supports a role for MTs in acquired drug resistance [27]. In most cell types, zinc is often sequestered through binding to MTs, keeping free zinc concentrations fairly low that could account for lack of function in a typical zinc-sensitive protein, such as p53 [28]. Indeed, zinc supplementation to HIPK2-depleted cells restores p53 native conformation and transcriptional activity in response to drugs, as well as increases *in vivo* tumor regression in combination with anticancer drug adryamicin (ADR) in a xenograft colon cancer cell model [22]. The finding of p53 misfolding upon HIPK2 depletion was corroborated by *in vivo* studies in mice with the transgenic MMTV-*neu* spontaneous breast cancer model that revealed low HIPK2 gene expression in the tumor tissue compared to normal tissue, that correlated with misfolded p53 [29]. Zinc treatment in combination with anticancer drug adryamicin remarkably reduced spontaneous tumor growth compared to drug treatment alone, restoring wild-type p53 (wtp53) conformation and p53 apoptotic transcriptional activity [29].

Among the regulators of the HIPK2-p53 signaling axis in response to DNA damage is the LIM (Lin-11. Isl-I and Mec3) domain protein Zyxin, a regulator of the actin skeleton and focal adhesions, that stabilizes HIPK2 by inhibiting Siah-1-mediated HIPK2 degradation [30]. Depletion of Zyxin, therefore, inhibits HIPK2 stabilization and DNA damage-induced p53Ser46 phosphorylation and apoptosis. Another molecule that fine-tunes the p53 activation threshold in response to differing severities of genotoxic stress is Axin that allows distinct complexes formation of p53 with molecules Pirh2, Tip60 and HIPK2 [31]. Under sublethal DNA damage, Pirh2 abrogates Axin-induced p53Ser46 phosphorylation by competing with HIPK2 for binding to Axin. Under lethal DNA damage Tip abrogates Pirh2-Axin binding forming an Axin-Tip60-HIPK2-p53 complex that allows p53 apoptotic activation [31].

### HIPK2 regulates molecules involved in p53-dependent and -independent apoptosis in response to genotoxic damage

HIPK2 promotes apoptosis by modulating factors, directly or indirectly related to p53, such as the antiapoptotic transcriptional corepressor CtBP [7], the p53 inhibitor MDM2 [32] and ΔNp63α [33]. HIPK2 participates in a pathway of UV-triggered CtBP clearance that results in cell death. HIPK2 phosphorylates CtBP at Ser-422 that induces protein degradation. Thus, HIPK2 knock-down inhibits UV-induced CtBP-Ser-422 phosphorylation and degradation in p53-null H1299 lung cancer cells, confirming HIPK2 role in apoptosis also in cells lacking p53 [7,34]. MDM2 is the main p53 negative regulator, it is an oncogene often upregulated in tumors and for these reasons many studies are devoted to the development of small molecules to inhibit MDM2 and restore p53 activity [11,35]. HIPK2, by phosphorylating MDM2 for proteasomal degradation [36], may overcome the MDM2-induced p53 inactivation and restore p53 apoptotic activity [32]. On the other hand, an intriguing regulatory circuitry between MDM2 and HIPK2/p53 axis revealed that sublethal DNA damage leads to HIPK2 inhibition by a protein degradation mechanism involving p53-induced MDM2 activity [37]. These findings highlight a role for MDM2 to fine-tune the p53-mediated biological outcomes (that is, cell cycle arrest *vs* apoptosis) according to cell requirement. However, this also explains the p53 inactivation in tumors overexpressing MDM2, nonetheless the presence of wtp53. In this latter case, the use of the small molecule RITA (reactivation of p53 and induction of tumor cell apoptosis) that inhibits MDM2/p53 interaction and induces expression of p53 target genes and massive apoptosis in various tumor cells lines [35], can be useful to counteract HIPK2 degradation and to reactivate p53 apoptotic function [38]. Interestingly, also zinc ions treatment has been shown to relapse the MDM2-induced HIPK2 downregulation, by counteracting the MDM2 E3 ubiquitin ligase activity finally reactivating the HIPK2-induced p53Ser46 phosphorylation and apoptotic activity [39], although the molecular mechanism needs to be elucidated.

HIPK2 depletion has been shown to induce cancer cell resistance to different anticancer drugs even in p53-null cells, suggesting the involvement of additional HIPK2 targets other than p53. In particular, it has been found that HIPK2 phosphorylates and promotes proteasomal degradation of ΔNp63α, a prosurvival dominant negative (DN) isoform of the p53 family member p63. HIPK2 phosphorylates ΔNp63α at the T397 residue, thus, the nonphosphorylatable ΔNp63α-T397A mutant is not degraded in spite of either HIPK2 overexpression or ADR treatment. These findings underline ΔNp63α as a novel HIPK2 target in response to genotoxic drugs [33]. These data indicate that HIPK2 has a double commitment, working as activator for proapoptotic factors (*i.e.*, p53) on one hand and inhibitor for antiapoptotic factors (*i.e.*, CtBP, MDM2, ΔNp63α, HIF-1α) on the other hand. On the opposite side, these considerations would allow to suppose that tumor-associated inhibition of HIPK2 activity might strongly contribute to chemoresistance and tumor progression, in addition to other better-characterized events, such as p53 mutation/inactivation and MDM2 or ΔNp63α overexpression.

### Mechanisms of HIPK2 inhibition and its impact on both p53 function and tumor progression

**Figure 1:**
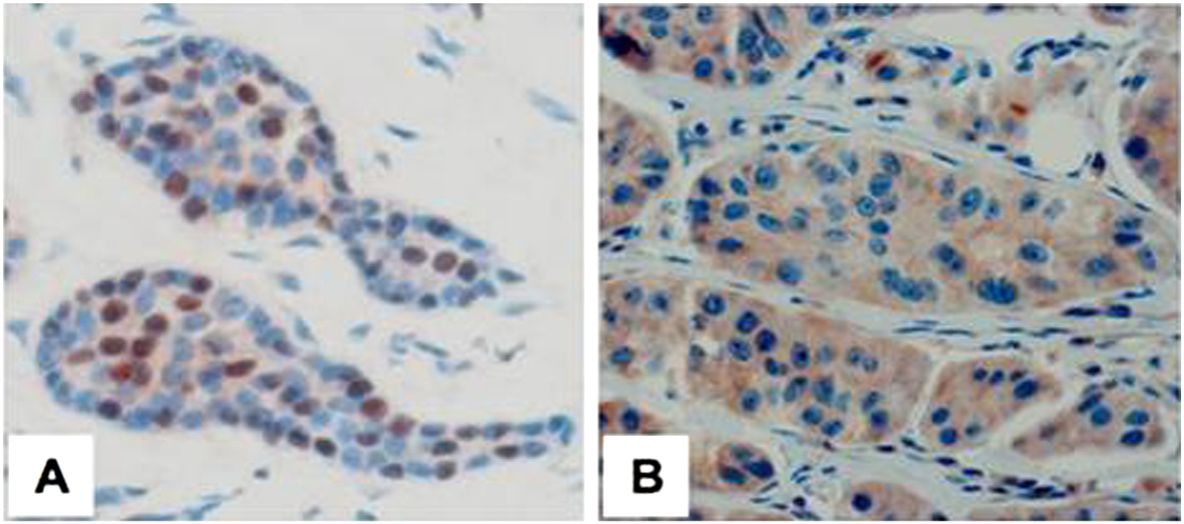
**HIPK2 immunostaining in breast cancer.** Streptavidin-biotin immunoperoxidase staining of invasive breast ductal carcinomas displaying (**A**) nuclear HIPK2 localization, and (**B**) cytoplasmic HIPK2 localization. Magnification 40X. (kindly provided by Dr. Marcella Mottolese, IFO-IRE, Rome, Italy).

Several proteins have been shown to target the HIPK2/p53 axis and therefore to inhibit stress- or drug-induced apoptosis to clear cancer. Recent studies demonstrated that High-mobility group A1 (HMGA1) proteins interact with p53 and inhibit its apoptotic activity [40]. Interestingly, HMGA1 overexpression is responsible for HIPK2 cytoplasmic sequestration and the subsequent inhibition of HIPK2/p53 interaction and apoptosis activation [41]. HMGA1 is frequently overexpressed in tumors and correlates with low apoptotic index in wild-type p53 breast cancer tissues [41]. Thus, immunostaining of breast ductal carcinomas with low HMGA1 expression and with high apoptotic index (not shown) results in HIPK2 nuclear localization (Figure 1A). On the other hand, breast ductal carcinomas with high HMGA1 expression and with low apoptotic index (not shown) show HIPK2 cytoplasmic localization (Figure 1B), meaning likely HIPK2 inactivation [41]. Similar HIPK2 cytoplasmic localization was found in breast cancer tissues with overexpression of integrin alpha(6)beta(4), which is also involved in impairment of p53 apoptotic activity [42], indicating that HIPK2 cytoplasmic relocalization may be a negative marker for p53 function.

HIPK2 is involved in the p53-mediated repression of Galectin-3 (Gal-3), a β-galactoside-specific lectin with anti-apoptotic activity, involved in tumorigenesis and resistance to chemotherapeutic drugs [43]. Intriguingly, though, Gal-3 is highly expressed in well-differentiated thyroid carcinomas (WDTCs) nonetheless the presence of wild-type p53 supposed to negatively regulate Gal-3. This paradoxical behavior may be explained by hypothesizing that in WDTC wtp53 protein is inactive. Thus, Real-Time PCR on total RNA extracted from frozen thyroid tissues samples as well as immuonohistochemistry analyses revealed that HIPK2 is indeed downregulated in WDTCs [44]. In particular, genetic loss at HIPK2 locus 7q32-34 was found by loss of heterozigosity (LOH) analysis in thyroid cancer cells stained with Gal-3 and retrieved by Laser Capture Microdissection (LCM) [44]. This study demonstrates that the loss of HIPK2 expression in WDTC may be responsible for lack of p53 activation, thus explaining the paradoxical co-expression of wild-type p53 with overexpressed Gal-3. Of interest, HIPK2 LOH was also observed in mice. In particular, a screening for genetic alterations in radiation-induced thymic lymphomas demonstrated that *Hipk2* is a haploinsufficient tumor suppressor gene *in vivo*, showing loss of one *Hipk2* allele in 30 % of the tumors and increased susceptibility of *Hipk2+/−* mice to radiation-induced thymic lymphoma [45]. This study provides compelling evidence that *Hipk2* functions as major tumor suppressor in response to ionising radiation *in vivo*. Interestingly, this function appears to be in part independent of p53.

An intact p53 is crucial for chemotherapy-induced apoptosis in MYCN-overexpressing neuroblastoma cells. Thus, MYCN sensitizes neuroblastoma cells to apoptosis by upregulation of the HIPK2/p53Ser46 pathway via ATM-dependent DNA damage response (DDR) that activates HIPK2 [46]. HIPK2 is largely expressed in human primary MYCN amplification (MNA) neuroblastoma tissues and its expression is induced by MYCN, whose inactivation inhibits HIPK2 and impairs p53Ser46 phosphorylation and apoptosis [46].

An abnormal HIPK2 function was recently associated to skin carcinogenesis. This study investigated a link between oncogene E6 of genital high-risk human papillomavirus (HPV) and HIPK2. The authors found that only E6 of beta2PV types (HPV23 and HPV38), but not beta1PV types (HPV8 and HPV20), cutaneous gammaPV (HPV4) or genital HPV16, physically interacts with HIPK2, inhibiting HIPK2-mediated p53Set46 phosphorylation by enforcing dissociation of the HIPK2/p53 complex [47]. This is relevant because HPV infection of keratinocytes prevents UV-activated cell death and thus may contribute to skin carcinogenesis, suggesting a possible mechanism that is inhibition of the HIPK2/p53 function. This finding highlights the role of HIPK2 as tumor suppressor that is in line with the outcome of genetic HIPK2 deletion in mice where *Hipk2−/−* and *Hipk2+/−* mice are tumor prone and undergo skin carcinogenesis by the two stage carcinogenesis protocol, showing that HIPK2 acts as a tumor suppressor in the skin [48]. The molecular mechanism was identified in increased Wnt/β-catenin-mediated cyclin D1 target gene expression, which is involved in cell proliferation. Thus, HIPK2 forms a protein complex with β-catenin and recruits the corepressor CtBP for cyclin D1 repression [48]. Subsequent studies demonstrated that HIPK2 phosphorylates β-catenin for proteasomal degradation [49], thus interfering with the transcription of several β-catenin target genes, including vascular endothelial growth factor (VEGF) involved in tumor angiogenesis and tumor growth [50].

Few mutation were also found in human acute myeloid leukemias (AMLs), which lead to aberrant HIPK2 nuclear distribution with impairment of p53 apoptotic transcriptional activity [51], confirming the role of HIPK2 in p53 activation to counteract tumor growth. However, additional studies are needed to evaluate the incidence of HIPK2 mutations in tumors.

**Figure 2:**
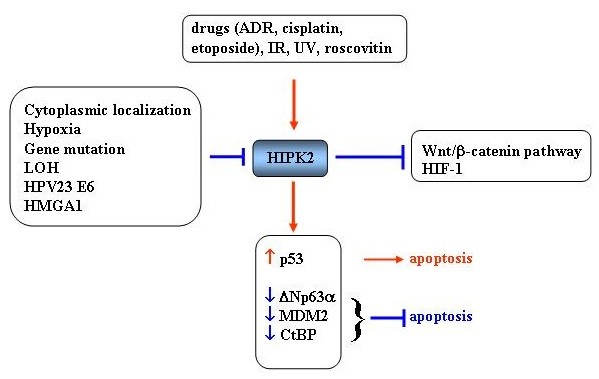
**Schematic representation of HIPK2 activation/inactivation.** HIPK2 can be activated by: drugs, IR, UV, roscovitin. The so far known mechanisms of HIPK2 inhibition are: cytoplasmic localization, hypoxia, gene mutation, LOH, and HPV23 E6 or HMGA1 overexpression. HIPK2 inhibits the oncogenic Wnt/β-catenin and HIF-1 pathways. HIPK2 activates p53 for apoptotic function and inhibits the antiapoptotic CtBP, MDM2 and ΔNp63α proteins.

A physiological condition that inhibits HIPK2 functions in solid tumor is hypoxia [52], a hallmark of tumor progression and failure of tumor therapies. Hypoxia activates the RING family ligase seven in absentia homolog-2 (Siah-2) that induces HIPK2 proteasomal degradation [52]. The presence of hypoxia renders tumor cells resistant to conventional chemo- and radiotherapy selecting a more malignant and invasive phenotype and plays a negative role in patient prognosis [53]. The key mediator in response to decreased oxygen availability is the transcription factor hypoxia-inducible factor-1 (HIF-1) that induces genes involved in angiogenesis, chemoresistance, glucose metabolism, and invasion. HIF-1 consists of the constitutively expressed HIF-1β subunit and the HIF-1α subunit, whose stability is stimulated by low oxygen or genetic alterations [53]. In this regard, it has been shown that HIPK2 represses HIF-1α gene transcription [54] counteracting the hypoxic phenotype and restoring tumor cell chemosensitivity in tumor cells irrespective of the *TP53* gene status [55]. Restoration of tumor cell chemosensitivity was also reported in another study showing that exogenous HIPK2 overexpression was able to circumvent inhibition of apoptosis in cisplatin-resistant ovarian cancer cells [56] although the molecular mechanism is still elusive. HIF-1α overexpression has been shown to antagonize p53-mediated apoptosis [57] also because HIF-1α may induce HIPK2 proteasomal degradation therefore inhibiting p53Ser46 phosphorylation [58]. This novel regulatory circuitry between HIF-1α, HIPK2 and p53 molecules gives a mechanistic explanation of the p53 apoptotic inhibition in response to drug under hypoxia in those tumors that retain a nonfunctional wild-type p53 [58]. Interestingly, HIF-1α may be targeted by zinc ions that induce HIF-1α proteasomal degradation [59], opening a way to reactivate the hypoxia-inhibited HIPK2/p53 pathway that could be exploited *in vivo*. This finding was corroborated by cDNA microarray studies in hypoxia-treated cancer cells, showing that zinc ions indeed reverse the hypoxia-induced gene transcription [60]. In summary, several different mechanisms that inhibit HIPK2 in tumors were identified, leading mainly to impairment of p53 response to drugs but also to induction of oncogenic pathways important in tumor progression, angiogenesis and chemoresistance such as Wnt/β-catenin and HIF-1 (Figure 2). During hypoxia, HIPK2 can be reactivated by zinc treatment that becomes a valuable tool to be used in combination with anticancer drugs to restore the HIPK2/p53 pathway.

### A novel role of HIPK2 in controlling cytokinesis and preventing tetraploidization

**Figure 3:**
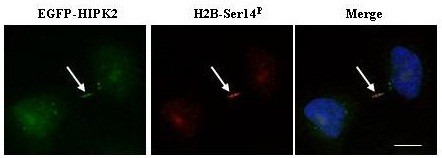
**HIPK2 and H2B-Ser14P co-localization at midbody.** HeLa cells were transfected with Flag-HIPK2 expression vector and immunostaining was performed with anti-Flag (green) and with anti phospho-Histone2B-Ser14 (H2B-Ser14P, red) antibodies. White arrows show midbody. Merge shows HIPK2 and H2B-Ser14P co-localization at midbody. Bar is 10 micron.

**Figure 4:**
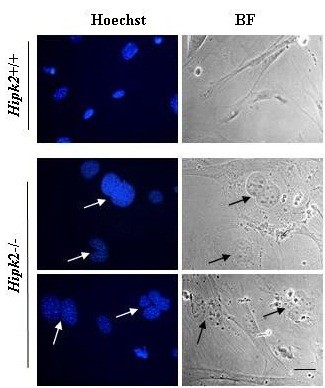
**HIPK2 knockout induces bi- and multi-nucleation.** Mouse embryo fibroblasts (MEFs) were obtained by wild-type (Hipk2+/+) and knockout (Hipk2-/-) mice. Cell nuclei were stained with Hoechst. Arrows indicate bi- and- multi-nucleated cells. BF: bright field. Bar is 10 micron.

Recently, an unexpected subcellular localization and biological function of HIPK2 in cytokinesis was identified [61]. In cytokinesis daughter cells separate by constriction of the cytoplasmic intercellular bridge between the two re-forming nuclei at the final step of cell division. Failure of cytokinesis may generate tetraploid cells. With the exception of rare cell types, such as hepatocytes, which can exist as stable tetraploids, tetraploid cells have chromosome unstable state that can lead to aneuploidy and ultimately to tumorigenic transformation [62]. Alike several abscission’s regulatory and effector components, HIPK2 and its novel target, the histone H2B, was shown to localize within the intercellular bridge at the midbody during cytokinesis. HIPK2 binds directly histone H2B and phosphorylates it at serine residue 14 (Ser14). Despite the apoptotic functions of both HIPK2 and the S14 phosphorylated form of H2B (H2B-S14^P^), the two proteins co-localize at the midbody (Figure 3), independently of the presence of chromatin in the cleavage plane, DNA damage, and/or apoptosis. HIPK2-depletion by targeted gene disruption or siRNA interference results in loss of H2B-S14^P^ at the midbody, impairs abscission, the final step of cytokinesis, and induces accumulation of cytokinesis-dependent aberrations, including tetra- and poly-ploidization (Figure 4). Remarkably, the expression of a phospho-mimetic H2B-S14D mutant can rescue these cytokinesis defects, showing that HIPK2-mediated H2B-S14 phosphorylation is required for a faithful cytokinesis [61]. This study suggests that HIPK2 may function as tumor suppressor also by preventing tetraploid cell formation and may have important implications to comprehend the mechanisms of safeguard from ploidy in which the p53 tumor suppressor is known to play important roles. Indeed, because of the key role of HIPK2 in p53 pro-apoptotic activation, HIPK2 inactivation may at once generate tetraploid cells and suppress their safety control. This latter statement is in agreement with a previous study showing that HIPK2 knockdown strongly abolished the tumor cell capacity to repair damaged DNA, at least in part through impairment of p53-function, suggesting that HIPK2 inhibition might increase genomic instability and thereby favor tumor progression [63]. In addition, the HIPK2-induced H2B activation reveals an unpredicted function of the extra-chromosomal activity of the H2B core histone, whose requirement for faithful cytokinesis can become a target for anti-cancer drugs. In future studies it would be interesting to evaluate in tumors the association between loss of HIPK2 function, H2B-S14 phosphorylation at the midbody and tetraploidy.

## Conclusion

In conclusion, the above summarized findings demonstrate how HIPK2 is important in inducing the apoptotic tumor response to genotoxic damage, and how is deeply involved in p53 regulation through different mechanisms including protein phosphorylation, acetylation, and protein conformation. HIPK2 may also indirectly affect p53 apoptotic function by modulating proteins involved in p53 deregulation such as Nox1, MT2A, MDM2, that are often upregulated in tumors and that account for tumor progression and chemoresistance. However, HIPK2 may induce apoptosis even in p53-null cells, downregulating for instance molecules such as antiapoptotic CtBP and ΔNp63α. These findings underscore how HIPK2 might affect several signaling pathways, including the oncogenic Wnt/β-catenin or HIF-1 pathways, involved in tumor progression and tumor response to therapies. They also underline the need to maintain an intact HIPK2 function. Among the mechanisms of HIPK2 inhibition it is lately becoming clear that hypoxia might negatively affect HIPK2 function and thereby indirectly also p53 oncosuppressor function. HIPK2 may undergo to some mutations, and another intriguing mechanism of HIPK2 inhibition is the reported LOH in well differentiated thyroid carcinomas and in mice. Moreover, the just discovered role of HIPK2 in cytokinesis implies its control on chromosomal instability which allows tumorigenesis. Therefore, these findings, by demonstrating the contributions of HIPK2 signaling to tumor regression and response to therapies, propose HIPK2 as potential diagnostic marker and a therapeutic target. What does the future hold for this promising tumor suppressor protein? Other than unveiling novel roles for HIPK2 in anticancer mechanisms, one intriguing area will be to discover selective compounds for HIPK2 (re)activation, for anticancer therapeutic purpose.

## Ethical approval

Any experimental research that is reported in the manuscript have been performed, reviewed, and approved by the appropriate ethics committee of the Regina Elena National Cancer Institute, Rome, Italy. Research carried out on humans was in compliance with the Helsinki Declaration, and the experimental research on animals followed internationally recognized guidelines.

## Abbreviations

ADR, Adryamicin; AMLs, Acute myeloid leukemias; DDR, DNA damage response; Gal-3, Galectin-2; HAT, Histone acetylase; HIF-1, Hypoxia inducible factor-1; HIPK2, Homeodomain-interacting protein kinase 2; HMGA1, High mobility group A1; HPV, Human papillomavirus; H2B, Histone H2B; IR, Ionizing radiation; LCM, Laser Capture Microdissection; LOH, Loss of heterozygosity; MNA, MYCN amplification; MT2A, Metallothionein 2A; Nox1, NADPH oxidase 1; RITA, Reactivation of p53 and induction of tumor cell apoptosis; siRNA, Small interference RNA; UV, Ultraviolet radiation; VEGF, Vascular endothelial growth factor; WDTC, Well differentiated thyroid carcinomas.

## Competing interests

The authors declare that they have no competing interests.

## Authors’ contributions

All named authors conceived the study, participated in its design and coordination and helped to draft the manuscript. All authors read and approved the final manuscript.
